# Effect of a High Protein, Low Glycemic Index Dietary Intervention on Metabolic Dysfunction-Associated Fatty Liver Disease: A Randomized Controlled Trial

**DOI:** 10.3389/fnut.2022.863834

**Published:** 2022-04-27

**Authors:** Ping Sun, Liping Huang, Ping Shuai, Zhengwei Wan, Yingying Liu, Jianqiang Xue, Yuping Liu

**Affiliations:** ^1^Department of Health Management and Institute of Health Management, Sichuan Provincial People's Hospital, University of Electronic Science and Technology of China, Chengdu, China; ^2^Chinese Academy of Sciences Sichuan Translational Medicine Research Hospital, Chengdu, China; ^3^Guangdong General Hospital, Guangdong Academy of Medical Sciences, Guangzhou, China

**Keywords:** high protein low glycemic index diet, MAFLD, NAFLD, dietary intervention, RCT

## Abstract

**Background:**

Metabolic dysfunction-associated fatty liver disease (MAFLD) affects people at an increasingly younger age. The primary treatment for patients with MAFLD is diet-induced weight loss; however, excessive dieting is poorly effective.

**Objectives:**

The aim of this trial was to evaluate whether a high protein and low glycemic index (HPLG) dietary intervention would result in improvement of controlled attenuation parameter (CAP) and related metabolic markers in MAFLD.

**Methods:**

A 12-week controlled, parallel-group, randomized intervention trial was performed. A number of 63 participants with MAFLD were enrolled and randomized between the HPLG dietary group and the balanced diet control group. Both diets had the same hypocaloric level and were prescribed *ad libitum* within food limit lists. The primary outcome was CAP. The main secondary outcomes were weight loss and improvement of metabolism-related indexes at week 12 after the program initiation.

**Results:**

A total of 59 participants completed the intervention and were included in the final analysis. The mean age was 39.3 ± 8.9 years and 66.1% were men. In this trial, protein and carbohydrate intakes were significantly higher and lower, respectively, in the HPLG group compared to controls (*p* < 0.001). At week 12, CAP was significantly reduced in both groups (*p* < 0.001). However, a significantly greater reduction in liver fat was observed in the HPLG group compared to the control group (*p* = 0.011), with mean relative reductions of 30.90 dB/m (95% CI, 21.53 to 40.26, *p* < 0.001) and 15.43 dB/m (95% CI, 7.57 to 23.30, *p* < 0.001), respectively. From baseline to week 12, a significantly greater loss in bodyweight was recorded in participants in the HPLG group (6.52 kg; 95% CI, 5.50 to 7.54, *p* < 0.001) compared to control subjects (2.00 kg; 95% CI, 0.89 to 3.11, *p* = 0.001). Moreover, body fat percentage in the HPLG group was significantly reduced compared with the control group (*p* = 0.002). Within-group improvements in visceral fat, blood pressure, cardiovascular risk factors, and blood glucose-related indicators were detected in patients with MAFLD assigned to the HPLG diet (*p* < 0.05), but not in those prescribed the control diet (*p* > 0.05).

**Conclusion:**

Under our experimental conditions, and compared to the traditional balanced diet, an HPLG diet led to a significant CAP remission, bodyweight or fat reduction, and improvement of metabolic markers in patients with MAFLD.

**Clinical Trial Registration:**

ClinicalTrials.gov, identifier: NCT03972631.

## Introduction

Metabolic dysfunction-associated fatty liver disease (MAFLD), a newly proposed term for non-alcoholic fatty liver disease (NAFLD), is currently the most common liver disease (1). MAFLD has a morbidity rate of 29.62% in the Asian population, representing a major challenge to the public health. The prevalence and impact of MAFLD in China vary greatly across different regions. For example, MAFLD-related morbidity in Shanghai is 38.17%, whereas in Chengdu is 12.5% (2, 3). MAFLD is usually associated with one or more chronic diseases that reduce life quality and increase mortality risk over time (4–7); hence, it imposes an ever-greater burden on the health of older adults. Current recommendations for managing MAFLD place a strong emphasis on the use of medications and bariatric surgery in middle and advanced stages, but provide less advice regarding early-stage interventions (1). This type of approach results in a considerable economic burden for patients and society.

Treatment of MAFLD focusing on weight loss through lifestyle modifications such as caloric restriction and exercise has proved to be of significant benefit (8, 9). Unfortunately, however, in the implementation of dietary interventions for MAFLD, limited attention has been paid to the role of diet type and nutritional composition, and the effect of excessive dieting showed to be poor. Although some studies addressed dietary patterns such as high protein and low glycemic index (HPLG) diets, the individuals involved had low rates of type 2 diabetes mellitus (T2DM) (10, 11). However, meta-analyses and randomized controlled trials (RCTs) have shown that diets that combine HPLG foods can reduce body fat and help weight maintenance in adults (12, 13). Consuming a higher proportion of energy from protein-rich foods combined with a low glycemic index dietary pattern optimizes nutrient composition without increasing total energy intake, which may improve dietary quality. Consequently, this approach appears to be sufficiently effective and may provide proper long-term adherence levels for Chinese patients with MAFLD. Nevertheless, there is limited evidence from high-quality trials evaluating the effects of HPLG diets on MAFLD, and more studies are needed to reinforce evidence-based dietary recommendations on the use of HPLG regimens.

We hypothesized that an HPLG dietary intervention is effective for the improvement of MAFLD, mainly through significant reduction of hepatic steatosis as assessed by controlled attenuation parameter (CAP) measurement. Thus, we conducted an RCT in patients with MAFLD to examine the effect of a 12-week intensive HPLG dietary intervention, using as control a balanced diet as recommended by the Dietary Guidelines for Chinese Residents. Our results provide preliminary guidance for designing effective nutritional interventions for MAFLD.

## Patients and Methods

### Subjects

This study was done in the Health Management Center of the Sichuan Provincial People's Hospital from August 2020 to November 2020. All the study participants provided written informed consent. The study protocol was in accordance with the ethical guidelines of the Declaration of Helsinki 1975, has been approved by the Human Ethics and Research Ethics committees of Sichuan Provincial People's Hospital (approval number 2019–311), and was registered in the ClinicalTrials.gov registry (NCT03972631).

### Inclusion and Exclusion Criteria

Eligible participants were between 18 and 65 years of age and reported a diagnosis of MAFLD (supported by available medical records). The study subjects had not met the standard of drug treatment as judged by their clinicians, had CAP scores > 240 dB/m, BMI between 25.0 and 35.0 kg/m^2^, originated from the Southwest region of China, and resided in Sichuan. MAFLD is diagnosed based on histological (biopsy), imaging, or blood biomarker evidence of hepatic fat accumulation (hepatic steatosis) in combination with one or more of the following three criteria: overweight or obesity, T2DM, or metabolic disorder (defined as having two or more risk factors for metabolic abnormalities) (14).

Individuals were excluded based on the current or previous (3 months before the study) use of hormonal or weight-affecting drugs; diseases that affect food digestion and absorption (such as chronic diarrhea, constipation, severe digestive tract inflammation, active digestive tract ulcer, post-gastrointestinal resection, and cholecystitis/post-cholecystectomy); tertiary hypertension, diabetes, anemia, and serious cardiovascular and cerebrovascular diseases; viral liver disease, autoimmune liver disease, cirrhosis, and other severe liver diseases; abnormal renal metabolism or kidney diseases that require control of protein intake; psychiatric diseases, memory disorders, epilepsy, use of anti-schizophrenia or anti-depression drugs; disabilities, cancer, or infectious diseases such as tuberculosis and AIDS; and contraindications for FibroTouch examination, such as pacemaker use, pregnancy, gestation, and lactation. The enrolled patients were screened and provided written informed consent. Final eligibility was determined prior to the participants being randomly assigned to one of the two study groups.

### Study Design and Outcomes

We conducted a randomized, parallel-group, prospective, 12-week controlled trial in MAFLD subjects. The main outcome was the CAP score at the end of the study, determined by FibroTouch. Secondary outcomes consisted of the following: (1) anthropometry: weight, waist circumference (WC), body fat percent; (2) liver function tests: alanine aminotransferase (ALT), aspartate aminotransferase (AST), gamma-glutamyl transferase (GGT); (3) cardiovascular risk (CVR) markers: relative brachial-ankle pulse wave velocity (baPWV), ankle-brachial pressure index (ABI), systolic blood pressure (SBP), diastolic blood pressure (DBP), homocysteine (Hcy); (4) lipids: total cholesterol (TC), triglyceride (TG), low-density lipoprotein cholesterol (LDL-C), high-density lipoprotein cholesterol (HDL-C); (5) fasting plasma glucose (FPG), fasting insulin (FINS), glycosylated hemoglobin (HbA1c), and homeostasis model assessment of insulin resistance (HOMA-IR).

### Randomization and Masking

Randomization was conducted by a researcher who was not involved in this trial, using a computer-produced sequence that allocated eligible individuals to randomly receive either the HPLG dietary intervention or a traditional, balanced diet intervention. Once assigned, a study identification number was provided to the participants. The nature of the interventions made it impossible to conceal participants and investigators (researchers and dietitian).

### Dietary Intervention

After randomization, the HPLG participants were asked to follow a HPLG carbohydrate diet (40–45% protein, 20–25% carbohydrate, 30–35% fat) with restricted energy content: initial bodyweight × 25 kcal/kg × 0.7. The calorie-restricted formula was based on the Consensus of Experts on Medical Nutrition Therapy for Overweight/Obesity in China (2016). Participants went through a 12-week phase of replacing the staple food with nutrition bars (provided at no cost to participants), followed by a 15-day balanced diet reintroduction phase. During the 12-week intervention, a detailed list of recommended HPLG subsidiary food was also provided. The nutrition bar was a HPLG product prescribed as an alternative to the staple food consumed daily at lunch and dinner; its formulation aimed to reduce carbohydrate, while assuring an adequate protein intake. Nutritional information for the supplement bars is shown in Supplementary Table 1. If required, a fiber supplement was recommended for constipation. When entering the final half-month meal transition phase, a traditional balanced diet pattern was recommended according to the Dietary Guidelines for Chinese Residents (2016). Over the transition period, calorie intake returned to normal levels: initial bodyweight × 25 kcal/kg. In this stage, consumption of nutrition bars was decreased by half a bar per week, staple food was reintroduced gradually, and a dietitian supervised the participants' meals.

Participants in the control group were provided with a traditional balanced diet (10–20% protein, 50–65% carbohydrate, 20–30% fat) according to the Dietary Guidelines for Chinese Residents (2016) with restricted energy content: initial bodyweight × 25 kcal/kg × 0.7. Thus, caloric restriction was the same as in the HPLG group. There was no transition period in the control group after the 12-week intervention. The intervention plan and range of food lists of the two dietary patterns described above are shown in Supplementary Tables 2, 3. Caloric content for each listed food can be found in the food bank of the dietary intervention mini-program (version 2.21, Zhejiang Notte Health Technology Co., Ltd, Hangzhou, China).

During the intervention, subjects were instructed to record their daily diet and exercise activities; these were monitored by the researchers and a dedicated dietitian, who conducted online supervision and gave follow-up recommendations as required. Accessing the dietary intervention mini-program, subjects recorded each meal's composition through photographs combined with text descriptions. Participants in the HPLG group were asked to have their urine ketone tested every morning from the 3rd day of intervention and upload the test results to the intervention mini-program. Project researchers and the dietitian monitored all data uploaded during the weight management phase to the dietary intervention platform using a mobile phone application or an online management system (NUTRIEASE 8.9.12, Notte, China). The study's dietitian assessed subjects' problems on a weekly basis through the intervention mini-program and gave further dietary guidance and suggestions according to the diet and weight changes reported. Furthermore, the study's dietitian contacted the participants by phone weekly, to provide dietary management support and assess trial compliance. Every 4 weeks, the researchers interacted with the participants at planned inspection visits. In addition, unscheduled consultations with researchers could be requested by the patients at any time. Both study groups were advised to arrange the type and amount of exercise according to their preferences. All MAFLD medications were discontinued 1 week prior to the trial initiation. Based on the health status of participants and upon clinical judgment, antihypertensive medications were dropped or adjusted.

This trail provided dietary and activity advice, as well as MAFLD education. Participants in both groups received support from a dedicated, trained dietitian, and from a clinical research coordinator and physicians that adhered to standard procedures for nutrition education interventions. The multidisciplinary team discussed the progress of participants to make the intervention consistent. Education and dietary prescription consisted of standardized dietary summaries and a list of food groups. Based on the dietary models and individual needs, suitable options were specified by estimating food amount and portion sizes. This trial provided also a detailed list of meal recipes specific to each diet. Regardless of intervention type, all subjects were provided with the equivalent level of treatment in terms of contact possibilities, type and quantity of written resources, number of suitable food choices, and individual advice on diet.

### Assessment

Throughout the study and during monthly scheduled visits, subjects who underwent the assessment of fasting biochemistry completed a standardized 3-day dietary questionnaire, a physical activity questionnaire, and anthropometric measures were taken. A diet history interview, conducted by a registered dietitian experienced in dietetic interventions, was used to record food intake and food composition data. Nutritional components were analyzed using an online nutritional analysis software (NUTRIEASE 8.9.12, Notte, China). Based on the meal pictures uploaded by the participants and information contained in the software's database, the study's dietitian broke down and quantified foods on the backstage management system. Before commencing the trial, participants completed also a standardized epidemiological questionnaire, which collected basic personal information, personal and family history of illnesses such as diabetes, hypertension, dyslipidemia, smoking, alcohol intake, medication status, and physical activity level. The questionnaire was filled out by uniformly trained researchers through face-to-face interviews. Throughout the trial, physical activity was evaluated using the modified International Physical Activity Questionnaire (long-form) (15). The type, intensity, frequency, and duration of exercise were recorded. The participants were suggested to keep their regular activity level during the study.

Height and body mass were measured using a Physical Examination Scale (HNH-219; Omron, Tokyo, Japan). Flexible steel girth tape was used to measure WC at the narrowest point between the edge of 10th rib and the iliac crest. The midpoint was used if narrowing was not clear. A medical automatic electronic sphygmomanometer (HPP-9020) was used to evaluate resting blood pressure (BP) after relaxed sitting. Each assessment used the average of two valid values.

Controlled attenuation parameter for liver fat and liver stiffness (LS) data was quantified using a Hisky Medical Technology's Fibrotouch-FT3000 device. During measurements, subjects were placed in supine position with the physician on their right side. After instructing the patients to raise their right hand behind their head, the probe was placed between the 7th and 9th costal spaces from the right axillary front to the midaxillary line, close to the intercostal space. A number of three anatomical points were selected to measure liver fat attenuation parameters. The median value of 10 successful measurements made at each of the above areas was finally selected (16). The test was consistently performed by a well-trained, experienced sonographer. CAP values are expressed as dB/m and reflect liver fat content (hepatic lipid). The principle of this examination is that the transmission of ultrasonic waves through the body will be affected (absorbed, scattered, or reflected) according to the fat content, resulting in the different degrees of signal attenuation. In the assessment of hepatic steatosis, scattering occurs when the incident ultrasonic wave encounters lipid droplets contained in hepatic cells. This results in increased ultrasonic attenuation, which correlates with the severity of fatty liver disease (17, 18). According to the standards outlined in the Expert Consensus on Clinical Application of Transient Elastography (TE) and based on the liver fat attenuation results detected by FibroTouch, patients with MAFLD were diagnosed as having mild fatty liver (CAP 240–265 dB/m), moderate fatty liver (CAP 265–295 dB /m), or severe fatty liver (CAP > 295 dB/m) (19). The MAFLD remission was defined as CAP values lower than the baseline value, whereas MAFLD elimination was defined as CAP <240 dB/m.

For all participants, 10-h fasting blood samples were obtained both at baseline and on week 12 and sent to the Clinical Laboratory of Sichuan Provincial People's Hospital for the analysis according to unified standards. The determinations included standard liver function tests: ALT, AST, and GGT; lipids: TC, TG, HDL-C, and LDL-C; blood glucose and insulin sensitivity indicators: FPG, FINS, HbA1c, HOMA-IR (measured as FPG × FINS/22.5); and CVR indicators: Hcy.

Ankle-brachial pressure index and baPWV were measured by the arteriosclerosis diagnostic device (Omron Colin BP-203RPEIII; VP-1000). Traditional risk factors of CVR include age, gender, BP, cholesterol, diabetes, and smoking. Hypertension is the most significant and common modifiable risk factor for early-onset cardiovascular disease (CVD) (20), and the study has shown that atherosclerosis is an independent predictor of cardiovascular events in hypertensive patients (21). Pulse wave velocity (PWV) is not only a surrogate marker of atherosclerotic load and an independent risk factor for CVD but can also be used to judge the efficacy of intervention. baPWV is more suitable for large-scale population epidemiological studies, where it proved to be an independent predictor of cardiovascular events and death (22, 23), and can be used as a biomarker of CVR in asymptomatic individuals (24). For population screening, ABI was also shown to be a strong predictor of future cardiovascular event risk (25). ABI and PWV should be tested simultaneously to better assess the patients' clinical conditions. These tests were performed by a single operator under standardized conditions. The result parameters were ABI and average baPWV relative to peers.

Bioimpedance analysis to measure body fat percent and limb skeletal muscle mass was carried out using an InBody 770 analyzer (InBody Co., South Korea), with subjects strictly following the voice prompts of the instrument.

A bioelectrical impedance analysis device (DUALSCAN, HDS-STD; Omron, Japan) was used to measure abdominal visceral fat and subcutaneous fat area. The visceral fat area was determined by the two parameters of abdominal width and biological resistance. Dedicated technicians operated the instrument according to the standard procedures.

### Statistical Analysis

Sample size calculations were carried out in PASS 15.0.5 software (NCSS, Kaysville, UT, USA) based on the data from Properzi et al. (26). To find an expected difference of 25% in MAFLD remission between groups after the 12-week intervention, considering a significance level of 5%, power of at least 80%, and allowing for 10% dropout, the study aimed to recruit 60 subjects. At least 27 subjects from each dietary group were required to detect diet-induced differences.

The case report form (CRF) was strictly reviewed by the quality control team and entered into the management system after confirmation. All follow-up data were recorded and time checked before being entered into the system.

At the end of the study, endpoints were analyzed based on the intention-to-treat method. IBM SPSS Statistical Analysis Software for Windows, version 23.0, was used for statistical analysis. After baseline values were adjusted, differences in macronutrient and clinical indicator outcomes between groups were examined using repeated measures analysis of covariance (ANCOVA). The Fisher's permutation test was used to assess the independence of the main outcome of this trial. Paired *t*-tests or nonparametric Wilcoxon signed-rank tests were used to analyze statistical differences within groups. The significance was determined using a *p-*value threshold of 0.05.

## Results

**Figure 1:**
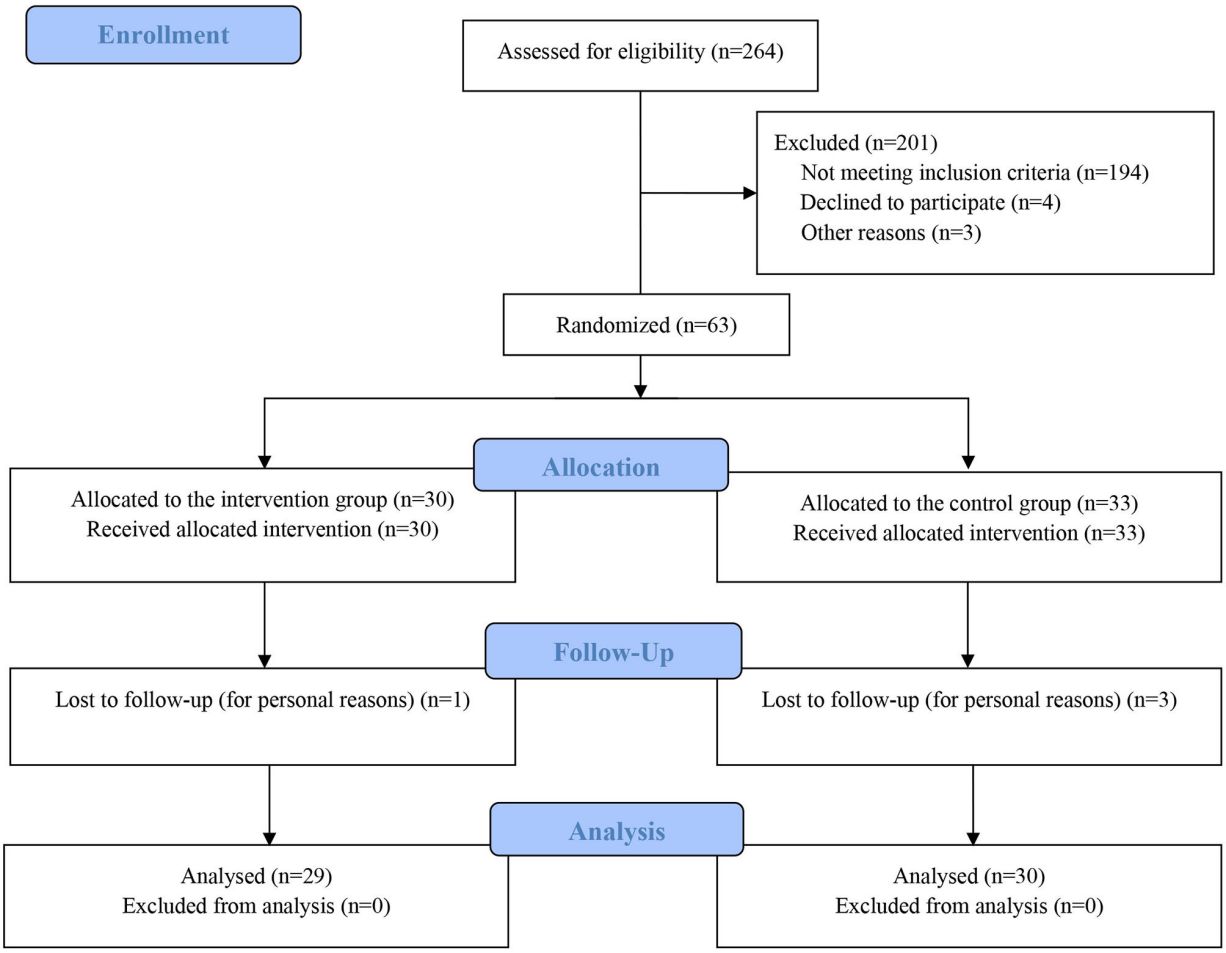
Study how chart showing the process of patient selection and enrollment, allocation to the two study groups, and rate of patients completing the study. HPLG group, high-protein and low glycemic index dietary regimen; Control group, conventional balanced dietary regimen.

Figure 1 shows a flow chart of study participation. A total of 264 patients with MAFLD were enrolled in this trial and were screened by the research fellows. This study registered 63 participants and they were attributed randomly to the HPLG diet group (*n* = 30) or the balanced diet control group (*n* = 33). A number of four (6.3%) participants withdrew from the study. Final analysis excluded the data from these participants and the final intention-to-treat analysis included a total of 59 subjects.

### Baseline Characteristic of Trial Participants

Baseline characteristics of the two groups were similar except that subjects in the HPLG group had significantly higher SBP (*p* = 0.002) and DBP (*p* = 0.003) (Table 1). For all subjects, the average age was 39.3 years (SD 8.9) and BMI was 28.4 kg/m^2^ (SD 2.6). The majority of participants (39; 66.1%) were men. Mean SBP was 124.3 mmHg (SD 12.8), mean DBP was 77.5 mmHg (SD 10.0), and 4 participants (6.8%) had hypertension. Average total cholesterol was 5.1 mmol/L (SD 1.0), and 19 study subjects (32.2%) had hyperlipidemia. Mean HbA1c was 5.38% (SD 0.35). Only 3 (5.1%) participants had a history of impaired fasting glucose (IFG). A number of eight (13.6%) participants were current smokers and 32 (54.2%) were current drinkers. No subjects had cirrhosis. The exercise volume reported by participants was 58.5 MET-h/week (SD 39.3).

**Table 1:** Baseline demographics and characteristics of the study groups.

	**Variables**	**HPLG**	**Control**	**Significance (*P*)**
Demographics	Age (years)	39.8 (9.6)	38.9 (8.3)	0.714
	Male (%)	19 (65.5)	20 (66.7)	0.926
Lifestyle	Smoker (%)	5 (17.2)	3 (10.0)	0.666
	Drinker (%)	16 (55.2)	16 (53.3)	0.887
	Activity (MET-h/week)	47.3 (78.3)	42.0 (41.6)	0.495^*^
Comorbidities (%)	IFG	0 (0)	3 (10.0)	0.248
	Hyperlipemia	10 (34.5)	9 (30.0)	0.713
	Hypertension	3 (10.3)	1 (3.3)	0.580
Anthropometry	Height (cm)	167.9 (8.5)	164.9 (8.5)	0.187
	Weight (kg)	80.7 (9.2)	76.8 (11.3)	0.152
	BMI (kg/m^2^)	28.6 (2.5)	28.1 (2.8)	0.379^*^
	WC (cm)	92.6 (5.8)	90.7 (8.8)	0.141^*^
	Body fat (%)	32.7 (6.1)	34.0 (7.6)	0.303^*^
	Skeletal muscle (kg)	30.4 (4.8)	28.4 (5.4)	0.126
	Visceral fat (cm^2^)	97.0 (26.5)	93.7 (33.7)	0.678
	Subcutaneous fat (cm^2^)	256.4 (45.1)	246.4 (61.5)	0.351^*^
CV indicators	Systolic BP (mm Hg)	129.4 (12.5)	119.5 (11.3)	**0.002**
	Diastolic BP (mm Hg)	81.3 (11.0)	73.7 (7.4)	**0.003**
	ABI	1.03 (0.07)	1.05 (0.07)	0.377
	Relative baPWV	15.1 (13.9)	11.9 (13.9)	0.384
	Hcy (μmol/L)	13.1 (4.8)	12.5 (4.0)	0.814^*^
Liver	CAP (dB/m)	292.3 (18.8)	291.0 (23.6)	0.810
	LS (kPa)	6.5 (1.6)	6.3 (1.5)	0.649^*^
	ALT (U/L)	51.5 (30.3)	47.2 (27.4)	0.509^*^
	AST (U/L)	33.9 (12.0)	31.9 (11.9)	0.490^*^
	GGT (U/L)	43.6 (26.9)	43.1 (37.6)	0.476^*^
Lipids	Total cholesterol (mmol/L)	5.1 (1.0)	5.1 (0.9)	0.879^*^
	TGs (mmol/L)	2.8 (2.3)	2.3 (2.0)	0.225^*^
	HDL-cholesterol (mmol/L)	1.3 (0.2)	1.2 (0.3)	0.170^*^
	LDL-cholesterol (mmol/L)	3.0 (0.8)	3.1 (0.6)	0.550
	FPG (mmol/L)	5.3 (0.6)	5.2 (0.6)	0.482
	FINS (μU/ml)	10.8 (5.1)	9.3 (5.1)	0.313^*^
	HbA1c (%)	5.4 (0.4)	5.4 (0.3)	0.964^*^
	HOMA-IR	2.6 (1.3)	2.2 (1.5)	0.243^*^

*Data presented as mean (SD) or number (percentage). Bold type indicates p < 0.05*.

* *Non-normal distribution. Normality determined using the Shapiro–Wilk test (p < 0.05). Non-normal distributions were analyzed for differences between groups using the Mann–Whitney U test. MET, metabolic equivalent; kPa, kilopascals*.

### Dietary Intervention

At baseline, total daily energy, macronutrient, and dietary fiber intakes were not significantly different between the HPLG and control groups (Table 2). Between groups or within each group, dietary status was significantly changed at week 12 (Table 2). Macronutrient intakes measured in both groups were close to those expected according to the study diets. Across the two groups, by the study's end, total energy intake was not significantly different, whereas macronutrient and fiber intakes differed significantly. Specifically, intakes of protein, fiber, and fat were higher, while carbohydrate intake was lower, in the HPLG relative to the control group.

**Table 2:** Nutrient intakes at baseline and completion.

							**HPLG (Baseline**	**Control (Baseline**
**Variables**	**Baseline**			**Week-12**			**vs. Week 12)**	**vs. Week 12)**
	**HPLG**	**Control**		**HPLG**	**Control**
	**(*n* = 29)**	**(*n* = 30)**	**Significance**	**(*n* = 29)**	**(*n* = 30)**	**Significance**	**Significance**	**Significance**
	**M (SD)**	**M (SD)**	**(*P*)**	**M (SD)**	**M (SD)**	**(*P*)**	**(*P*)**	**(*P*)**
Energy (kcal)	1,374 (388)	1,248 (351)	0.212	1,251 (251)	1,168 (264)	0.463	0.172^*^	**0.039**
Protein (g)	64.4 (19.8)	56.3 (20.0)	0.137	105.2 (27.6)	63.1 (16.6)	**<0.001**	<0.001^*^	**<0.001**
Carbohydrate (g)	137.1 (44.9)	127.4 (44.4)	0.426	68.9 (19.2)	108.4 (36.2)	**<0.001**	<0.001^*^	**<0.001**
Fat (g)	58.8 (19.1)	57.0 (19.7)	0.920^*^	64.5 (13.7)	53.7 (12.2)	**0.004**	0.165^*^	0.677
Fiber (g)	9.1 (5.7)	7.0 (3.8)	0.073^*^	11.6 (4.9)	6.1 (2.1)	**<0.001**	0.032^*^	0.319^*^
% energy from	19.1 (3.8)	18.2 (3.9)	0.378	34.0 (5.0)	22.0 (4.5)	**<0.001**	<0.001^*^	**<0.001**
protein
% energy from	42.3 (8.6)	41.6 (8.2)	0.773	19.8 (5.0)	36.9 (6.3)	**<0.001**	<0.001^*^	**<0.001**
carbohydrate
% energy from	38.8 (7.0)	40.3 (6.8)	0.292^*^	46.2 (4.5)	41.3 (4.2)	**<0.001**	<0.001^*^	**<0.001**
fat

* *Non-normal distribution. Bold type indicates p < 0.05. Normality determined using the Shapiro–Wilk test (p < 0.05). Non-normal distributions were analyzed for differences between groups using the Mann–Whitney U test*.

### CAP, LS, and Liver Biochemistry

Between- and within-group changes in physical activity, anthropometric, and biochemistry parameters are shown in Tables 3, 4, respectively. Compared to the baseline values, CAP decreased significantly in both groups (*p* < 0.001), with reductions of −30.9 dB/m (SD 24.6) in the HPLG group and −15.4 dB/m (SD 21.1) in the control group. At the completion and after baseline adjustment, CAP in the HPLG group was significantly lower than in the control group (*p* = 0.011). Significant differences in CAP variation were also found after adjusting for changes in body fat percent (*p* = 0.01) or weight (*p* = 0.03). At the end of treatment, the two groups showed no significant differences in AST and LS. In the HPLG group LS, AST, ALT, and GGT levels were significantly reduced after the intervention, while in the control group, LS and AST did not change significantly.

**Table 3:** Comparison of endpoint results between groups adjusted for baseline values.

**Variable**	**HPLG diet**	**Control**	**Significance (*P*)**
Lifestyle			
Activity (MET-h/week)	66.4 (38.1)	60.8 (37.3)	0.904
Anthropometry			
Weight (kg)	74.1 (7.8)	74.8 (11.7)	**<0.001**
BMI (kg/m^2^)	26.3 (2.3)	27.4 (2.1)	**<0.001**
Waist (cm)	84.8 (5.5)	87.5 (8.8)	**0.003**
Body fat (%)	28.6 (6.7)	32.1 (7.7)	**0.002**
Skeletal muscle (kg)	29.7 (4.6)	28.2 (5.3)	0.062
Visceral fat (cm^2^)	73.0 (30.8)	83.6 (35.9)	0.050
Subcutaneous fat (cm^2^)	197.0 (43.6)	228.4 (68.5)	**<0.001**
CV indicators			
Systolic BP (mm Hg)	120.6 (11.5)	118.0 (11.1)	0.164
Diastolic BP (mm Hg)	73.8 (10.1)	74.0 (9.6)	**0.038**
ABI	1.06 (0.08)	1.05 (0.07)	0.429
Relative baPWV	7.8 (10.7)	9.5 (14.7)	0.233
Hcy (μmol/L)	10.7 (2.3)	11.5 (3.3)	**0.046**
Liver			
CAP (dB/m)	261.4 (25.8)	275.6 (25.9)	**0.011**
			**0.01** ^†^
			**0.03** ^‡^
LS (kPa)	5.9 (1.1)	5.8 (1.3)	0.943
ALT (U/L)	24.3 (11.5)	31.2 (16.4)	**0.009**
AST (U/L)	27.2 (12.6)	28.5 (9.8)	0.562
GGT (U/L)	22.0 (11.4)	32.7 (27.8)	**<0.001**
Lipids			
Total cholesterol (mmol/L)	4.95 (0.89)	4.96 (0.96)	0.985
TGs (mmol/L)	1.57 (1.63)	1.67 (2.10)	0.288
HDL-cholesterol (mmol/L)	1.23 (0.24)	1.18 (0.19)	0.657
LDL-cholesterol (mmol/L)	3.00 (0.67)	3.07 (0.64)	0.983
FPG (mmol/L)	5.14 (0.71)	5.09 (0.61)	0.714
FINS (μU/ml)	6.89 (3.40)	7.84 (3.75)	**0.024**
HbA1c (%)	5.22 (0.43)	5.33 (0.33)	0.256
HOMA-IR	1.63 (0.93)	1.83 (1.10)	**0.045**

*Data presented as mean (SD) or median (interquartile range). Bold type indicates p < 0.05. MET, metabolic equivalent; kPa, kilopascals*.

† *Significance determined using Fisher's permutation test by adjusting for the change in body fat percentage*.

‡ *Significance determined using Fisher's permutation test by adjusting for the change in weight*.

**Table 4:** Within-group changes from baseline to end of study.

		**Baseline:**	**Week 12:**	**Significance**	**Baseline:**	**Week 12:**	**Significance**
	**Variables**	**HPLG Diet**	**HPLG Diet**	**(*P*)**	**Control**	**Control**	**(*P*)**
Lifestyle	Activity (MET-h/week)	62.4 (38.5)	66.4 (38.1)	0.344^*^	54.7 (40.4)	60.8 (37.3)	0.368^*^
Anthropometry	Weight (kg)	80.7 (9.2)	74.1 (7.8)	**<0.001**	76.8 (11.3)	74.8 (11.7)	**0.001**
	BMI (kg/m^2^)	28.6 (2.5)	26.3 (2.3)	**<0.001**	28.1 (2.8)	27.4 (3.1)	0.002^*^
	WC (cm)	92.6 (5.8)	84.8 (5.5)	**<0.001**	90.7 (8.8)	87.5 (8.8)	0.009^*^
	Body fat (%)	32.7 (6.1)	28.6 (6.7)	**<0.001**	33.9 (7.7)	32.1 (7.7)	0.001^*^
	Skeletal muscle (kg)	30.4 (4.8)	29.7 (4.6)	**<0.001**	28.4 (5.4)	28.2 (5.3)	0.274
	Visceral fat (cm^2^)	97.0 (26.5)	73.0 (30.8)	0.001^*^	90.5 (31.3)	83.6 (35.9)	0.165
	Subcutaneous fat (cm^2^)	256.4 (45.1)	197.0 (43.6)	<0.001^*^	248.8 (61.7)	228.4 (68.5)	**0.008**
CV indicators	Systolic BP (mm Hg)	129.4 (12.5)	120.6 (11.5)	**<0.001**	119.5 (11.3)	118.0 (11.1)	0.428
	Diastolic BP (mm Hg)	81.3 (11.0)	73.8 (10.1)	**<0.001**	73.7 (7.4)	74.0 (9.6)	0.866
	ABI	1.03 (0.07)	1.06 (0.08)	0.077	1.05 (0.07)	1.05 (0.07)	0.803
	Relative baPWV	15.1 (13.9)	7.8 (10.7)	**0.001**	11.8 (14.1)	9.5 (14.7)	0.369^*^
	Hcy (μmol/L)	13.1 (4.8)	10.7 (2.3)	0.001^*^	3.91 (0.71)	3.29 (0.60)	0.226^*^
Liver	CAP (dB/m)	292.3 (18.8)	261.4 (25.8)	**<0.001**	291.0 (23.6)	275.6 (25.9)	**<0.001**
	LS (kPa)	6.5 (1.6)	5.9 (1.1)	0.026^*^	6.3 (1.5)	5.8 (1.3)	0.091^*^
	ALT (U/L)	51.5 (30.3)	24.3 (11.5)	<0.001^*^	47.2 (27.4)	31.2 (16.4)	<0.001^*^
	AST (U/L)	33.9 (12.0)	27.2 (12.6)	0.009^*^	31.9 (11.9)	28.5 (9.8)	0.178^*^
	GGT (U/L)	43.6 (26.9)	22.0 (11.4)	<0.001^*^	43.1 (37.6)	32.7 (27.8)	0.001^*^
Lipids	Total cholesterol (mmol/L)	5.09 (1.00)	4.95 (0.89)	0.343	5.11 (0.92)	4.96 (0.96)	0.347^*^
	TGs (mmol/L)	2.82 (2.29)	1.57 (1.63)	<0.001^*^	2.27 (1.99)	1.67 (2.10)	0.007^*^
	HDL-cholesterol (mmol/L)	1.29 (0.24)	1.23 (0.24)	0.169	1.20 (0.25)	1.18 (0.19)	0.597
	LDL-cholesterol (mmol/L)	2.96 (0.80)	3.00 (0.67)	0.757	3.08 (0.65)	3.07 (0.64)	0.916
	FPG (mmol/L)	5.34 (0.63)	5.14 (0.71)	**0.036**	5.22 (0.65)	5.09 (0.61)	0.181
	FINS (μU/ml)	10.8 (5.1)	6.9 (3.4)	**<0.001**	9.34 (5.15)	7.84 (3.75)	0.041^*^
	HbA1c (%)	5.36 (0.36)	5.22 (0.43)	**0.008**	5.40 (0.34)	5.33 (0.33)	0.399^*^
	HOMA-IR	2.6 (1.3)	1.6 (0.9)	<0.001^*^	2.24 (1.46)	1.83 (1.10)	0.057^*^

*Data presented as mean (SD) or median (interquartile range). Bold type indicates p <0.05*.

* *Non-normal distribution. Significance levels derived from the Wilcoxon signed-rank test. MET, metabolic equivalent; kPa, kilopascal*.

### Anthropometry and Physical Activity

At the study's end, bodyweight, BMI, and WC decreased significantly in both groups. Weight loss in the HPLG group was greater than in the control group [6.5 (2.7) vs. 2.0 (3.0) kg, respectively; *p* < 0.001), reflecting respective reductions of 8.1 and 2.6% from baseline. Likewise, WC reduction was significantly greater in the HPLG group than in the control group (*p* = 0.003). After the intervention, fat mass percentage was significantly reduced in the HPLG group compared to the control group (*p* = 0.002). Skeletal muscle mass was significantly decreased within the HPLG group but did not differ between the two groups and within the control group. Physical activity did not differ significantly between groups at baseline and did not change significantly upon trial completion.

### Cardiovascular Risk

At the study's end, DBP and Hcy were significantly lower in the HPLG group, while SBP, ABI, and relative baPWV showed no difference between groups. Within groups, BP, relative baPWV, and Hcy were significantly improved in the HPLG group, but not in the control group. Arterial stiffness, as estimated by ABI, showed no difference within and between groups.

### Other Metabolism-Related Indicators

Lipid analyses showed that upon the study completion, plasma TG was significantly reduced in both groups, whereas total cholesterol, HDL-C, and LDL-C showed no significant variation. Moreover, no intergroup differences were observed in fasting lipids measures at week 12.

Relative to baseline, HOMA-IR and HbA1c improved significantly in the HPLG group (*p* < 0.05 for both variables), but not in the control group. In turn, at the end of the intervention IR, but not HbA1c, values for HPLG participants were significantly lower than those recorded for the control group (*p* = 0.045).

### Adverse Event Report

A number of four subjects in the control group reported two adverse events, constipation and arthritis. A number of three subjects in the HPLG group reported constipation, one diarrhea, two fatigue, one lymph node inflammation, one frequent flatulence, and one abdominal pain. In both groups, all adverse events were mild. Most adverse events in the HPLG group were potentially associated with the high-protein diet and resolved with appropriate management.

## Discussion

The present clinical trial was designed to investigate whether an HPLG diet can alleviate clinical symptoms of MAFLD and improve MAFLD-related anthropometric and biochemical markers. The study proved that compared with a conventional balanced diet, an HPLG diet can significantly improve liver function and metabolism-related indicators in patients with MAFLD. Of note, both bodyweight and body fat percentage were significantly reduced after 12 weeks of the intervention. The extent of weight loss, up to 10% relative to baseline, was higher than the 3–5% reduction required to significantly improve fatty liver (27). This finding adds to mounting evidence that dietary intervention, as a primary treatment for MAFLD, is effective for weight loss.

Our study showed that patients with MAFLD, identified by FibroTouch, can change their diet to follow an HPLG dietary pattern over a 3-month period. This dietary regimen significantly improved the fatty liver condition and body composition of the patients, which is consistent with the previous studies addressing the impact of this dietary pattern on other diseases (28). It is interesting to note that weight loss in study participants assigned to the HPLG diet was correlated with a significant reduction in body fat percentage, while skeletal muscle mass remained comparable to that recorded in control subjects. It is also worth noting that in the intervention group, liver fat and body fat varied independently. We observed no change in physical activity levels during the intervention period, suggesting, as discussed below, that weight loss depended on factors other than exercise expenditure.

High protein intake promotes earlier satiety and also enhances or prolongs satiety (29). The increased protein content in the HPLG diet may be the dietary component that prevented muscle levels from falling significantly below those of control participants during the weight loss process (30). Comparable to our findings, Larsen et al. reported that subjects on a high-protein diet (where ~25% of the energy comes from protein) lost more weight than those on a normal protein content diet or a low glycemic index diet alone (31).

Similar to the Larsen et al. and Jenkins et al. studies (31, 32), a significant reduction in carbohydrate energy supply ratio may have also contributed to the weight loss observed in the HPLG group. In addition, this group consumed high levels of polyphenols, which are found in low glycemic fruits and vegetables. These organic compounds have a wide range of benefits, including inhibiting liver lipid production, improving insulin sensitivity, and reducing CVD risk (33, 34). In view of the fact that CVD is the major cause of death in patients with MAFLD (6, 7, 35), this study suggests that medical nutrition therapy including a HPLG dietary pattern may help reduce MALFD-related, CVD-mediated mortality (36). In addition, consistent with related research (37–39), the reason that skeletal muscle mass in the HPLG group was modestly but significantly decreased after the 12-week intervention was probably related to body water weight loss due to the reduction in dietary carbohydrate and to negative energy balance, both of which would be expected to deplete glycogen and associated water. Moreover, given that numerous studies have reported an increase in FPG during a low glycemic diet intervention (40, 41), the fact that FPG and HbA1c remained basically unaltered in both groups was not surprising. Moreover, the control diet group consumed a relatively low amount of carbohydrates (108 g) every day. This may account for why more dramatic differences in outcomes between diets were not observed.

The greater loss of liver fat and bodyweight observed in the HPLG group may be related to the significantly increased dietary fiber intake associated with this diet. The study indicated that dietary fiber inhibits the absorption of cholesterol and improves the control of cholesterol levels (42). It has also been suggested that the increase of total fiber affects the gut microbiota, thereby impacting the intestinal-liver axis, which is related to the development and progression of fatty liver disease (43). In addition, by replacing high-carbohydrate foods with grains, tubers, and root vegetables, these diets are relatively high in fiber, resulting in a lower energy density intake. However, the part of the reason for the considerable loss of fat evidenced by trial participants may be due to timely and effective communication guidance provided by the dietitian, which motivated participants to regulate their appetite and adhere to and follow the diet plan.

During the trial period, both absolute lipid intake and percentage of lipid energy supply increased significantly in the HPLG group, which is consistent with a marked reduction in dietary carbohydrate and an increase in meat, eggs, and milk intake without strict control of lipid composition. The study has shown that saturated fat intake can increase liver fat content and liver IR (44). This may be the reason why no significant differences were detected in blood lipids such as TC, HDL-C, and LDL-C within the HPLG dietary group, and no significant differences in blood lipids, relative baPWV, FPG, and HbA1c between the HPLG and the control groups.

Detailed information about the subjects' daily diets was available on the study's diet registration platform. This enabled us to confirm dietary compliance in both groups, which supports the validity of our results. During the intervention period, we found a good diet adherence by the subjects in the HPLG group. The participants mostly expressed the satisfaction about the diet plan, but often lessened their commitment after ~4 weeks due to the intervention's positive effect. After this period, some participants went through a bottleneck period of weight loss. The compliance of the conventional diet group was mediocre. This may be due to the weight loss effect being not obvious by the fourth week of retest, and subjects were prone to burnout during the intervention process. In addition, two long Chinese holidays, National Day and Spring Festival, fell within the intervention period. This may have led some study subjects to temporarily deviate from the intervention protocol and might thereby affect the overall intervention effect in this study.

In the collection and analysis of all data, bias was addressed by assessing diet compliance through self-patient and investigator management; using the same dietitian and a randomized controlled intention-to-care design throughout the study helped minimize the possibility of selection bias. The bias was centered on the actual food choices of the patients. Given that both dietary intervention models had the same degree of bias, we assumed that the underlying bias was similar across groups. Considering the absence of exercise interventions, insignificant differences in activity levels among the groups were within the range of changes expected.

No previous dietary intervention for MAFLD has focused on the changes in body fat and muscle, nor presented data on dietary macronutrients, as we did in this study. In addition, the study had the advantage of determining the ability of participants to achieve the nutritional goals associated with an HPLG dietary pattern within the range of their personal food preferences. The wide selection of food banks and the implementation of dietary intervention requirements regardless of geographic location and living environment implied that results of this RCT can easily be translated into clinical prescriptions for medical nutrition therapy. Both dietary change and long-term adherence are the important factors for achieving meaningful results and reducing the costs of health care. Thus, our study provides solid evidence that an HPLG diet, as a clinical therapeutic tool, can be used in the context of usual food preferences and availability.

## Study Limitations

We made a comprehensive assessment of fatty liver and metabolism-related risk factors and monitored also potential confounders such as activity levels, which further enhanced the robustness of our results. However, similar to the previous diet studies, the short-term nature of the reported intervention may explain the reason why no more significant differences were found in outcomes in the HPLG group compared to the balanced diet group. More long-term and large-sample trials are needed to confirm whether an HPLG dietary pattern represents an effective treatment for MAFLD. Second, although widely used, the FibroTouch technique is not a well-accepted method to measure liver fat contents. Third, the loss of skeletal muscle measured by the body composition analyzer represented most likely water. Thus, changes in body protein and moisture should be respectively analyzed and discussed in further clinical trials. In addition, the energy supply distribution of macronutrients in the HPLG group did not strictly meet the requirements of the dietary intervention. Because dietary fat (along with protein and carbohydrate) contents differed in each diet, results of this study were likely influenced by the higher fat and lower carbohydrate content of the HPLG diet. Notwithstanding, longer and better efficacy of nutritional therapies for MAFLD may be associated with higher levels of dietary compliance and improved quality of life.

## Conclusions

This study demonstrated that a 12-week intervention pattern consisting of a HPLG diet resulted in varying degrees of improvement in fatty liver and metabolism-related indicators in patients with MAFLD. Compared to the balanced diet study group, the intervention led to significant weight loss, mainly due to a reduction in body fat, in overweight or obese adults.

## Data Availability Statement

The original contributions presented in the study are included in the article/Supplementary Material, further inquiries can be directed to the corresponding author.

## Ethics Statement

The studies involving human participants were reviewed and approved by the Human Ethics and Research Ethics Committees of Sichuan Provincial People's Hospital (Approval Number 2019–311). The patients/participants provided their written informed consent to participate in this study.

## Author Contributions

PSU conceived the study and designed the intervention. LH, ZW, PSH, YIL, JX, and YUL contributed to the study design. YUL was the clinical trial lead and supervised the study. PSH and ZW provided dietetic support. YIL and JX provided physician support and oversight. PSU and LH contributed to the data analysis and drafted the manuscript. All authors critically reviewed the manuscript and approved the final version.

## Funding

The study was supported by research grants awarded to YUL by the Science and Technology Department of Sichuan Province (2020YFS0557 and 19ZDYF0315) and the National Key R & D Program of China (2017YFC0113901).

## Conflict of Interest

The authors declare that the research was conducted in the absence of any commercial or financial relationships that could be construed as a potential conflict of interest.

## Publisher's Note

All claims expressed in this article are solely those of the authors and do not necessarily represent those of their affiliated organizations, or those of the publisher, the editors and the reviewers. Any product that may be evaluated in this article, or claim that may be made by its manufacturer, is not guaranteed or endorsed by the publisher.
