# Supplementary material for: Effect of a High Protein, Low Glycemic Index Dietary Intervention on Metabolic Dysfunction-Associated Fatty Liver Disease: A Randomized Controlled Trial
Source: Front Nutr. 2022 Apr 27;9:863834. doi: 10.3389/fnut.2022.863834 (PMC9097015; doi:10.3389/fnut.2022.863834)
Supplement: Supplementary file 1 [file Table_1.DOCX]

Supplementary Material

# Supplementary Tables

Supplementary Table 1. Nutrition Information of Staple Food Replacement Bar.

| Items | Per 100g | Nutrient Reference Value% |
| --- | --- | --- |
| Energy | 1418kJ | 17% |
| Protein | 31.2g | 52% |
| Fat | 14.3g | 24% |
| Trans Fatty Acids | 0g |  |
| Carbohydrate | 28.6g | 10% |
| Dietary Fiber | 4.9g | 20% |
| Sodium | 347mg | 17% |

Supplementary Table 2. Intervention Regimes.

|  | Food | HPLG Group | Control Group |
| --- | --- | --- | --- |
| Breakfast | staple food | 0 | 125g |
|  | egg | 50g | 50g |
|  | low-fat milk | 250ml | 250ml |
|  | vegetable | 100g | 0 |
|  | fruit | 150g | 100-200g (a.m.) |
| Lunch | staple food | 0 | 175g |
|  | nutrition bar | 1 | 0 |
|  | meat (cooked) | 200-300g | 150-250g |
|  | vegetable | 200g | 250g |
|  | fruit | 0 | 100-200g (p.m.) |
| Dinner | staple food | 0 | 125g |
|  | nutrition bar | 1 | 0 |
|  | meat (cooked) | 125-200g | 100-175g |
|  | vegetable | 200g | 250g |
|  | beans | 0 | 50g |
| Others | oil | 25g | 25g |

More than 1800ml drinking water is needed every day.

The number of nutrition bars should not be less than 2/day (unless the dietitian requires adjustment).

Dietitian should be informed in advance if subject not feeling well and using medicine.

Subjects need to consult dietitian in advance to obtain dietary adjustment plan before social engagement and holiday.

Maintain pre-intervention activity levels based on individual situation.

Supplementary Table 3. Range of Food Lists.

|  | HPLG Group | Control Group |
| --- | --- | --- |
| Meat | aquatic products  poultry (peeled chicken, duck and goose)  pure lean meat of livestock (pig, cattle and mutton) | aquatic products  poultry (peeled chicken, duck and goose)  pure lean meat of livestock (pig, cattle and mutton) |
| Vegetable | leafy vegetables  bacteria and algae  melons and eggplants (except pumpkin) | leafy vegetables  bacteria and algae  melons and eggplants (except pumpkin) |
| Fruit | low glycemic index fruits (cherry, grapefruit, pitaya, fresh peach, strawberry, orange, blueberry, etc.) | low glycemic index fruits (cherry, grapefruit, pitaya, fresh peach, strawberry, orange, blueberry, etc.) |
| Avoid | staple food (rice, noodles, etc.) starchy vegetables  legumes (kidney beans, beans, etc.)  animal offal  dessert, candy, ice cream, biscuits and other high sugar food  sugary drinks  all the wines | animal offal  dessert, candy, ice cream, biscuits and other high sugar foods  sugary drinks  all the wines |

1. The calorie level of each food can be found in the food bank of the dietary intervention mini-program.
2. Try to choose all foods within range.
3. Try to avoid cooking method with sugar, thickening and frying.
